# Intimate Partner Violence and Mental Well-Being Among Married Women Attending Psychiatric Outpatient Clinics: The Roles of Childhood Trauma and Self-Compassion

**DOI:** 10.3390/jcm15155809

**Published:** 2026-07-24

**Authors:** Zeynep Nur Demirok, Erdoğdu Akça, Mehmet Buğrahan Gürcan, Aybüke Yılmaz, Öznur Dilara Tüzün, Merve İpek, Merih Altıntaş

**Affiliations:** 1 Department of Psychiatry, Kartal Dr. Lutfi Kırdar City Hospital, 34865 İstanbul, Turkeymerveipekpsy@gmail.com (M.İ.); merihaltintas@yahoo.com (M.A.); 2 Department of Psychiatry, Marmara University School of Medicine, 34854 İstanbul, Turkey; erdogduakca@gmail.com

**Keywords:** childhood trauma, intimate partner violence, mental well-being, self-compassion, women

## Abstract

**Background/Objectives**: Intimate partner violence (IPV) is a public health problem associated with adverse mental health outcomes among women. Although prior research has focused on psychological distress (PD), mental well-being (MWB) is a distinct dimension of mental health that has received less attention in IPV research, particularly in clinical samples. Childhood trauma (CT) and self-compassion (SC) may contribute to variability in IPV-related outcomes. This study examined associations between IPV and MWB, considering SC, PD, and CT within an integrated framework. **Methods**: In this cross-sectional study, 340 married women aged 18–65 years attending psychiatric outpatient clinics completed self-report measures of IPV, CT, SC, PD, and MWB. Hierarchical regression analyses examined predictors of MWB, and conditional process analysis tested moderated serial mediation models evaluating indirect associations of IPV with MWB through SC and PD, with CT as moderator. **Results**: SC subscales accounted for the largest incremental variance in MWB (ΔR^2^ = 0.256 and 0.294, both *p* < 0.001), exceeding contributions of IPV and CT. CT moderated the IPV–SC association (B = 0.0052, 95% CI (0.0008, 0.0097), *p* = 0.022), but not the IPV–PD pathway. IPV showed indirect associations with MWB through SC and PD. **Conclusions**: These findings suggest that IPV-related mental health outcomes may be better understood within an integrative framework including vulnerability-related factors and psychological resources. Clinically, routine assessment of IPV, CT, SC, PD, and MWB together may help clinicians develop more comprehensive, trauma-informed and self-compassion-focused care plans for women in psychiatric outpatient settings.:

## 1. Introduction

Mental well-being (MWB) is a multidimensional construct that encompasses not only hedonic components such as happiness and life satisfaction, but also eudaimonic dimensions, including a sense of meaning, purpose, competence, and functional engagement in life [1]. From this perspective, MWB represents a broader and more integrative indicator of positive mental health rather than merely the absence of psychological symptoms. Individuals with higher levels of MWB demonstrate greater capacity for realizing their potential, coping effectively with everyday stressors, and experiencing psychological flourishing [2]. There is evidence that MWB is associated with life events and that these associations may differ by gender [3]. Among women, exposure to intimate partner violence (IPV) appears to be one of the critical life experiences associated with MWB [4,5].

IPV is defined as any behavior within an intimate relationship that causes physical, sexual, or psychological harm, including physical aggression, sexual coercion, psychological abuse, and controlling behaviors [6]. Women are at substantially higher risk of exposure to IPV than men, and IPV disproportionately affects women on a global scale [7]. Recent World Health Organization (WHO) estimates indicate that approximately one in three ever-partnered women have experienced physical and/or sexual IPV during their lifetime, while about 11% report exposure within the past 12 months [8,9]. Considering that psychological IPV is often reported to be even more prevalent than physical or sexual forms of violence [7], the overall prevalence of IPV is likely to be considerably higher when psychological violence is also taken into account.

Beyond its adverse effects on MWB, IPV has been consistently associated with a wide range of psychological and clinical outcomes [7]. Exposure to IPV has been shown to be associated with elevated psychological distress (PD) [10], as well as increased prevalence of common psychiatric conditions such as depression, anxiety disorders, and posttraumatic stress disorder among affected individuals [11,12].

According to the dual continua model of mental health, PD and MWB represent related yet distinct constructs [13]. A review of the literature indicates that research on IPV against women has predominantly focused on psychological distress outcomes, with comparatively fewer studies examining MWB. Only a limited number of studies have investigated PD and well-being concurrently [14].

Studies involving women exposed to IPV in adulthood have consistently identified adverse childhood experiences (ACE) as an important risk factor [15,16,17]. ACE may have long-lasting associations with emotion regulation, attachment patterns, and interpersonal functioning, which may contribute to vulnerability in later intimate relationships [16,17]. In addition, chronic childhood stress has been linked to alterations in developing stress-response systems and higher allostatic load, which may be associated with enduring mental and physical health risks in adulthood [18].

Mental health outcomes among individuals exposed to IPV are not homogeneous and show considerable individual variability, independent of the severity or type of violence. There is evidence suggesting that ACE may partly account for this heterogeneity in IPV-related mental health outcomes [19]. Consistent with this perspective, recent studies further indicate that childhood trauma (CT) may function as a moderating factor in the relationship between IPV and vulnerability-related psychological processes, suggesting that IPV-related psychological correlates may vary depending on individuals’ developmental trauma histories [20]. Accordingly, examining IPV, CT, and mental health outcomes together may provide a more comprehensive understanding of the developmental and relational dimensions of women’s mental health.

Efforts to explain the psychological correlates of IPV have increasingly extended beyond individuals’ developmental histories to include current internal psychological resources. Self-compassion (SC) refers to responding to one’s own suffering, failure, or inadequacy with kindness, understanding, and a sense of shared humanity rather than self-judgment [21]. In recent years, SC has emerged as a construct of growing interest in the context of IPV. The existing literature suggests that exposure to IPV may be associated with lower levels of SC [22]. At the same time, SC has been identified as a relevant factor in relation to both psychological outcomes and coping processes among IPV survivors. Studies have shown that higher SC is associated with fewer trauma-related symptoms, including posttraumatic stress, and with better psychological well-being [23]. In addition, SC has been linked to help-seeking behaviors among women exposed to IPV [24]. Preliminary pilot evidence in female survivors of IPV or gender-based violence further supports the clinical relevance of compassion-based approaches in this population [25]. Accordingly, enhancing SC in women exposed to IPV has been proposed as a potential intervention target for supporting psychological well-being and facilitating engagement with mental health services [26].

Although IPV has been widely studied, its clinical relevance in routine mental health settings remains insufficiently explored. Most research relies on community samples or women seeking help specifically for violence-related concerns. However, IPV among women presenting to psychiatric outpatient services with heterogeneous and non-specific complaints has received limited attention [27]. Mental health services may be particularly important settings for IPV assessment because IPV may remain hidden when services focus primarily on psychiatric symptoms rather than lived experiences and relational contexts. IPV may also overlap with depression, anxiety, trauma-related symptoms, and nonspecific psychological distress, while disclosure may be hindered by shame, fear of judgment or retaliation, stigma, safety concerns, or difficulties recognizing experiences as IPV. These considerations highlight the need for trauma-informed assessment and safe inquiry about IPV within mental health care [28]. At the same time, research on IPV in clinical mental health contexts has largely focused on psychopathology, emphasizing PD and trauma-related symptoms. MWB, however, represents a distinct dimension of mental health that cannot be inferred from symptom severity alone. Examining PD and MWB together therefore provides a more comprehensive perspective, particularly in clinical contexts.

Previous mediation studies have mainly focused on CT, identifying SC as a self-related psychological resource linking early adversity to both PD and MWB [29,30]. Extending this perspective, the present study considers SC within the context of ongoing relational adversity. Given its established associations with both distress-related symptoms and MWB, SC represents a conceptually relevant construct in this context. In the proposed serial pathway, SC was placed before PD for theoretical reasons: Drawing on evidence linking SC to trauma appraisals, emotion regulation, and posttraumatic symptoms, SC was conceptualized as a self-related psychological resource relevant to how IPV-related emotional distress is experienced [21,30], whereas PD was conceptualized as a more proximal indicator of current symptom burden [31]. Examining SC within indirect association models provides a coherent way to situate IPV within an integrated mental health framework that encompasses both vulnerability- and resource-oriented dimensions.

CT represents an additional contextual factor that may contribute to variability in IPV-related mental health outcomes, yet its role remains underexamined. Within this broader trauma context, SC may also be particularly relevant in psychiatric populations because difficulties in self-compassion have been described as potential barriers to trauma-focused treatment [32]. In such settings, SC is closely related to how trauma exposure and mental health coexist.

Together, these fragmented associations underscore the need for clinically grounded approaches that consider IPV, CT, SC, PD, and MWB within a single conceptual framework. The present study focused on women who had been legally married for at least one year in order to examine IPV within a legally and socially defined current intimate relationship framework and to reduce heterogeneity related to relationship status and duration. This design choice improved sample homogeneity but also narrowed the scope of the IPV construct by excluding dating, cohabiting, divorced, separated, and non-legally recognized relationships. Within this defined scope, the primary aim of this study was to examine the associations between IPV and MWB among married women presenting to psychiatric outpatient clinics, while simultaneously accounting for PD and SC in an integrated analytical framework. In addition, the study aimed to explore the role of CT as a contextual factor that may condition these associations. Because of the cross-sectional design, the proposed pathways were theoretically ordered but empirically associational, without implying causal or temporal sequence or excluding alternative directions of association. Given the limited empirical evidence on whether CT conditions the associations between IPV, SC, PD, and MWB, the hypothesis regarding the moderating role of CT was considered theory-driven and exploratory rather than firmly established.

Based on this rationale, the following hypotheses were tested:

1. SC will account for unique variance in MWB beyond the contributions of both CT and IPV.
2. SC will be positively associated with MWB and will be involved in the association between IPV and MWB, both independently and in sequence with PD.
3. CT will moderate the association between IPV and SC, such that the strength of this association will vary across levels of CT exposure.
4. CT will also moderate the association between IPV and PD, indicating variability in distress-related responses to IPV across different developmental backgrounds.

## 2. Materials and Methods

### 2.1. Study Design and Setting

This study employed an observational cross-sectional design and was conducted at the psychiatric outpatient clinics of Kartal Dr. Lütfi Kırdar City Hospital between December 2025 and February 2026. The study population consisted of women who had been legally married for at least one year and who presented to psychiatric outpatient clinics seeking mental health care for any reason during the study period.

A total of 340 married women were included in the study. Eligible participants were women aged 18–65 years who had been married for at least one year and provided written informed consent. Exclusion criteria comprised: (I) illiteracy; (II) a diagnosis of intellectual disability, specific learning disorder, neurocognitive disorder, or autism spectrum disorder; (III) meeting diagnostic criteria for alcohol or substance use disorder; and (IV) current or subsequent clinical diagnosis of bipolar disorder, schizophrenia spectrum disorder, or other psychotic disorders. Current alcohol consumption was recorded as a sociodemographic variable and did not constitute an exclusion criterion unless DSM-5 criteria for alcohol use disorder were met.

### 2.2. Sample Size

A priori power analyses were conducted using G*Power 3.1 (linear multiple regression, fixed model, R^2^ deviation from zero; α = 0.05, two-tailed; power = 0.80). Effect size assumptions were informed by prior literature examining associations among CT, IPV, SC, PD, and MWB. Previous studies have reported small to moderate proportions of explained variance for CT and IPV variables in multivariable models predicting mental health outcomes [33,34], whereas SC has shown moderate associations with MWB [35]. Accordingly, a conservative small-to-medium overall effect size (f^2^ = 0.10) was assumed for hierarchical regression models predicting MWB with up to 15 predictors, yielding a minimum required sample size of N = 201. For the moderated serial mediation framework, power was determined based on the component regression expected to have the smallest effect, namely the moderation [interaction] term. Consistent with prior methodological recommendations indicating that interaction effects are typically small [36], an effect size of f^2^ = 0.05 was specified for models including approximately eight predictors, resulting in a required sample size of N = 309. To account for potential data loss, missingness, and model complexity, the target sample size was increased by approximately 10%, and a final target of 340 participants was set.

### 2.3. Procedure

Participants were recruited using a convenience sampling approach from women attending the psychiatric outpatient clinics during the study period. Potentially eligible participants were approached after completion of their routine psychiatric evaluation and, when necessary, initiation or adjustment of treatment. Psychiatric diagnoses and exclusion criteria were assessed by psychiatrists through DSM-5-oriented routine clinical interviews. Eligible individuals were informed about the study procedures and invited to participate voluntarily, and written informed consent was obtained prior to data collection. A sociodemographic data form was then completed by the researchers, followed by self-report questionnaires completed by the participants, with assistance provided when required. The questionnaires were administered in paper-based format and completed in Turkish. Completion of the study forms took approximately 20–30 min, excluding the routine psychiatric evaluation. The questionnaires used in the study are described in detail in the following subsection.

Because intimate partner violence is a sensitive and potentially safety-relevant topic, additional precautions were taken during data collection. Questionnaires were completed in a private clinical setting without the presence of partners or accompanying relatives. Participants could skip any question they did not wish to answer. Confidentiality was maintained throughout the assessment, except in situations involving imminent risk of harm. When participants disclosed ongoing violence, acute safety concerns, suicidal ideation, or need for further support, they were evaluated by the psychiatrists conducting the assessment, and appropriate safety planning, crisis management, and referrals to institutional, psychosocial, or legal support services were provided in line with routine clinical procedures.

### 2.4. Study Measures and Questionnaires

#### 2.4.1. Intimate Partner Violence

Exposure to IPV was assessed using the Composite Abuse Scale (CAS), a comprehensive and multidimensional measure developed to evaluate IPV experiences during the preceding 12 months [37]. The CAS consists of 30 items rated on a frequency-based response and yields four subscale scores: Severe Combined Abuse, Emotional Abuse, Physical Abuse, and Harassment. The total score ranges from 0 to 150, with higher scores indicating greater exposure to abuse. The Turkish validity and reliability study of the CAS was conducted in 2025 and demonstrated satisfactory psychometric properties [38]. For the primary inferential analyses, CAS scores were treated as continuous variables: the four subscale scores were entered into the hierarchical regression analysis, whereas the total score was used to represent overall IPV exposure in the moderated serial mediation model. For descriptive purposes only, prevalence estimates were additionally calculated using the CAS manual’s total-score thresholds of ≥3 and ≥7 and the recommended subscale cutoffs. In the present study, internal consistency was acceptable to excellent across CAS subscales, with Cronbach’s α values ranging from 0.71 to 0.94, and high for the total scale (α = 0.94).

#### 2.4.2. Childhood Trauma

CT exposure was assessed using the Childhood Trauma Questionnaire (CTQ), originally developed by Bernstein et al., a 28-item measure that includes three items assessing minimization or denial of trauma and yields five subscale scores—emotional abuse, physical abuse, sexual abuse, emotional neglect, and physical neglect—as well as a total score [39]. The Turkish adaptation of the CTQ was validated by Şar et al. (2012), with evidence of acceptable reliability and validity [40]. In the present sample, internal consistency coefficients for CTQ subscales ranged from 0.70 to 0.91, and the total scale demonstrated acceptable reliability (α = 0.80).

#### 2.4.3. Self-Compassion

SC was measured using the Self-Compassion Scale–Short Form (SCS-SF), developed by Raes et al. (2011) as a brief version of the original Self-Compassion Scale [41]. The scale assesses six dimensions: self-kindness, self-judgment, common humanity, isolation, mindfulness, and over-identification. Isolation, over-identification, and self-judgment subscales were reverse-coded in the calculation of the total SCS score. The Turkish adaptation demonstrated satisfactory psychometric properties and was used in the present study [42]. In this study, Cronbach’s α values for SCS-SF subscales ranged from 0.70 to 0.87, with acceptable internal consistency for the total score (α = 0.81).

#### 2.4.4. Mental Well-Being

MWB was assessed using the Warwick–Edinburgh Mental Well-Being Scale–Short Form (WEMWBS-SF), which consists of seven items rated on a five-point Likert scale [43]. The scale measures positive aspects of MWB, including psychological functioning and affective states, with higher scores indicating greater well-being. The Turkish validity and reliability study was conducted by Demirtaş and Baytemir [44]. The scale demonstrated good internal consistency in the present sample (α = 0.84).

#### 2.4.5. Psychological Distress

PD was assessed using the Patient Health Questionnaire–4 (PHQ-4), an ultra-brief self-report screening tool developed by Kroenke et al. [31]. The PHQ-4 includes four items rated on a four-point Likert scale and yields a total score reflecting overall PD. Previous studies have demonstrated acceptable reliability and validity of the Turkish version [45]. In this study, the PHQ-4 showed acceptable internal consistency (α = 0.79).

### 2.5. Statistical Analyses

Analyses were conducted using IBM SPSS Statistics version 25.0 (IBM Corp., Armonk, NY, USA) and R (version 4.4.2). Descriptive statistics for all study variables were summarized using means, standard deviation, and observed ranges. Internal consistency was evaluated using Cronbach’s α.

Missing data were minimal (<2%) and handled in R using multiple imputation by chained equations (mice package) [46]. Ten imputed datasets were generated using predictive mean matching for continuous variables and tree-based methods for categorical or ordinal variables. Missingness diagnostics were consistent with a missing at random assumption. A single completed analysis dataset was then created by averaging continuous variables across imputations and assigning the modal category for categorical variables.

Primary analyses were performed using conditional process analysis with the PROCESS version 4.1 macro for SPSS (Model 84) [47]. All continuous variables were mean-centered prior to analysis. Indirect effects were estimated using 5000 bootstrap samples, and 95% percentile confidence intervals were reported. Conditional effects were examined at low (−1 SD), mean, and high (+1 SD) levels of the moderator. Because PROCESS does not directly pool conditional process models across multiply imputed datasets, analyses were conducted on the completed dataset; PROCESS estimates were therefore not pooled using Rubin’s rules. Complete-case sensitivity analyses were not conducted, as the missingness pattern was partly block-wise and complete-case analysis could have introduced non-random case exclusion and reduced power.

To further examine the relative contribution of subscale-level predictors, hierarchical multiple regression analyses were conducted, with changes in explained variance (ΔR^2^) used to evaluate incremental effects.

## 3. Results

### 3.1. Sociodemographic and Clinical Characteristics of Participants

The sample comprised 340 married women who presented to psychiatric outpatient services seeking mental health care, with a mean age of 39.67 years (SD = 9.48; range = 20–65). The mean duration of marriage was 15.90 years (SD = 10.24), and the mean age at marriage was 23.68 years (SD = 5.28). The largest proportion of participants had completed high school (32.6%), followed by those with a bachelor’s degree (20.9%). Approximately one third of the participants were employed. A lifetime psychiatric diagnosis was reported by 72.9% of the sample, and 28.0% reported a family history of psychiatric disorder. Smoking was reported by 42.1% of participants, whereas alcohol use was reported by 15.6%. Stressful life events in the last three months were reported by 55.0% of the sample. Based on DSM-5-based clinical assessments, the most common diagnosis at the time of evaluation was major depressive disorder, which was identified in 133 participants (39.1%). Anxiety disorders were the second most frequent diagnostic category (*n* = 105, 30.9%), followed by clinically significant subthreshold depressive and/or anxiety symptoms that did not meet full DSM-5 criteria for a specific depressive or anxiety disorder (*n* = 61, 17.9%). Less frequent diagnoses included obsessive–compulsive disorder (*n* = 20, 5.9%), trauma and stressor-related disorders, including adjustment disorder and posttraumatic stress disorder (*n* = 14, 4.1%), and somatic symptom disorder (*n* = 7, 2.1%). At the time of assessment, 184 participants (54.1%) were using psychotropic medication. Full descriptive statistics for sociodemographic and clinical variables are reported in Table 1.

### 3.2. Descriptive Statistics of the Measures

The CTQ total score had a mean of 42.41 ± 12.75 (24–88). Total CAS scores showed a mean of 10.42 ± 15.68 (0–122). The SCS total score averaged 35.79 ± 8.58 (16–56). The WEMWBS total score was 22.59 ± 5.82 (9–35). The PHQ-4 total score showed a mean of 7.08 ± 2.95 (0–12). Subscale-level descriptive statistics are provided in Table 2. Based on the CAS total-score cutoff of ≥3, 195 participants (57.4%) met the criterion for IPV exposure. When the more conservative cutoff of ≥7, recommended to minimize false-positive classifications, was applied, 39 participants (11.5%) met the criterion. The prevalence of emotional abuse, physical abuse, harassment, and severe combined abuse according to the corresponding CAS subscale cutoffs is presented in Table 1 [48].

### 3.3. Bivariate Correlations

Exploratory bivariate correlations indicated that several SC subscales were moderately associated with trauma indicators. The strongest associations were observed between isolation and CTQ emotional abuse (r = −0.32, *p* < 0.001), and between isolation and CTQ total score (r = −0.17, *p* = 0.001). Self-kindness showed a negative association with CTQ emotional abuse (r = −0.27, *p* < 0.001) and CTQ total score (r = −0.25, *p* < 0.001). Self-judgment was also negatively correlated with CTQ emotional abuse (r = −0.29, *p* < 0.001) and CAS emotional abuse (r = −0.29, *p* < 0.001). Other correlations between self-compassion subscales and trauma variables were smaller in magnitude.

MWB showed its strongest positive associations with overall SC (r = 0.56, *p* < 0.001), self-kindness (r = 0.47, *p* < 0.001), and mindfulness (r = 0.43, *p* < 0.001). In contrast, MWB was negatively associated with PD (r = −0.42, *p* < 0.001) and with CTQ emotional abuse (r = −0.27, *p* < 0.001). PD was most strongly correlated with CAS emotional abuse (r = 0.31, *p* < 0.001) and overall SC (r = −0.42, *p* < 0.001).

Age was positively associated with overall SC (r = 0.30, *p* < 0.001) and well-being (r = 0.20, *p* < 0.001), and negatively associated with PD (r = −0.28, *p* < 0.001). Duration of marriage showed a similar pattern, with positive correlations with SC total score (r = 0.29, *p* < 0.001) and MWB (r = 0.15, *p* = 0.006).

Bivariate correlations among the main study variables are presented in Table 3, while the full correlation matrix, including SC subscales, trauma subdomains, age, and duration of marriage, is provided as a correlation heatmap in Supplementary Figure S1.

### 3.4. Hierarchical Regression Analyses

Two separate hierarchical regression analyses were conducted to examine variables associated with MWB. Covariates were selected a priori on conceptual grounds. Age, income, education, and employment were included as relatively stable sociodemographic factors associated with IPV and mental well-being. Clinical variables were not additionally adjusted for because several may reflect current psychiatric burden, overlap with psychological distress, or lie within the pathways examined. Diagnostic heterogeneity, limited medication detail, and the close dependence of marriage duration on age further limited their suitability as covariates.

Regression assumptions were examined for both hierarchical regression models using residual histograms, P–P plots, residual-versus-predicted plots, Cook’s distance, leverage values, and multicollinearity indices. Residual diagnostics did not indicate severe deviations from normality or a clear heteroscedasticity pattern. Multicollinearity was not problematic in either model, with VIF values ranging from 1.092 to 2.844 in the CAS/IPV model and from 1.037 to 2.178 in the CTQ model. No observation exceeded the conventional Cook’s distance threshold of 1 in the CAS/IPV model, although 21 observations exceeded the conservative 4/N criterion; in the CTQ model, 18 observations exceeded the 4/N criterion. Maximum leverage values were 0.327 and 0.154 for the CAS/IPV and CTQ models, respectively, exceeding conventional leverage thresholds, particularly in the CAS/IPV model. Therefore, robust regression models using lmrob were additionally presented in the Supplementary Material as sensitivity analyses (Tables S1 and S2). The original CAS/IPV metric was retained to preserve the interpretability of IPV severity. Full model coefficients are reported in Table 4.

In Model 1, the block including CTQ subscales accounted for a significant increment in explained variance beyond the covariates (ΔR^2^ = 0.074, ΔF = 5.57, *p* < 0.001). Within this block, CTQ emotional abuse was negatively associated with MWB (β = −0.28, t = −3.90, *p* < 0.001). The subsequent inclusion of SC subscales resulted in a substantial additional increase in explained variance (ΔR^2^ = 0.256, ΔF = 22.18, *p* < 0.001). In the final model, MWB was positively associated with self-kindness (β = 0.25, t = 4.47, *p* < 0.001) and mindfulness (β = 0.19, t = 3.41, *p* = 0.001), and negatively associated with isolation (β = −0.26, t = −4.72, *p* < 0.001). No CTQ subscales remained significantly associated with MWB after the inclusion of SC variables.

In Model 2, the block comprising CAS subscales produced a modest but significant increase in explained variance (ΔR^2^ = 0.033, ΔF = 3.00, *p* = 0.019). None of the individual CAS subscales showed significant associations with MWB. Adding SC subscales yielded a marked increment in explained variance (ΔR^2^ = 0.294, ΔF = 25.42, *p* < 0.001). In the final model, MWB was significantly associated with self-kindness (β = 0.26, t = 4.83, *p* < 0.001), mindfulness (β = 0.19, t = 3.34, *p* = 0.001), and isolation (β = −0.26, t = −4.71, *p* < 0.001).

### 3.5. Moderated Mediation Analysis

**Figure 1:**
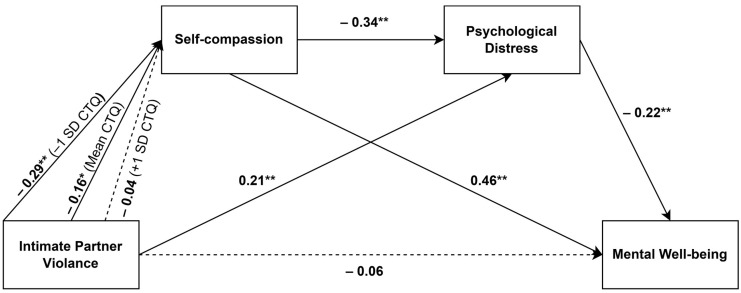
Conceptual moderated serial mediation model illustrating the hypothesized pathways linking intimate partner violence to mental well-being through self-compassion and psychological distress, with childhood trauma (CTQ) moderating the association between intimate partner violence and self-compassion. Solid arrows represent statistically significant paths, whereas dashed arrows represent non-significant paths.* *p* < 0.01, ** *p* < 0.001.

**Figure 2:**
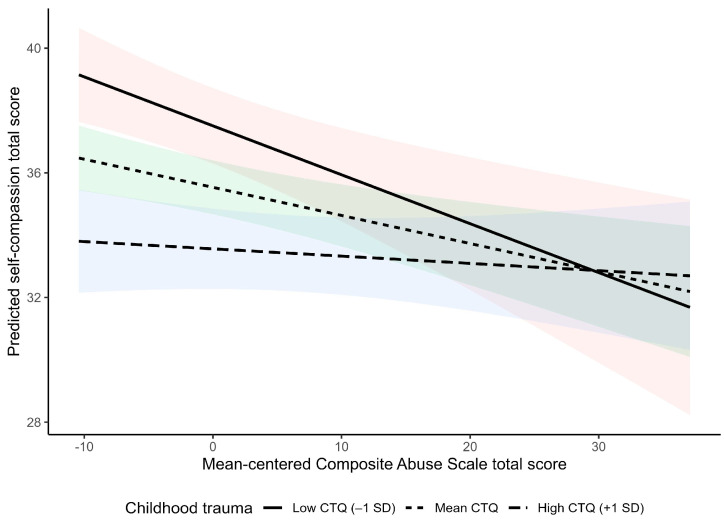
Simple Slopes of the Association Between Intimate Partner Violence and Self-Compassion Across Levels of Childhood Trauma. Predicted self-compassion scores are shown across mean-centered Composite Abuse Scale total scores at low (−1 SD), mean, and high (+1 SD) levels of Childhood Trauma Questionnaire total scores. Covariates were held at their sample means. Shaded areas represent 95% confidence intervals.

**Figure 3:**
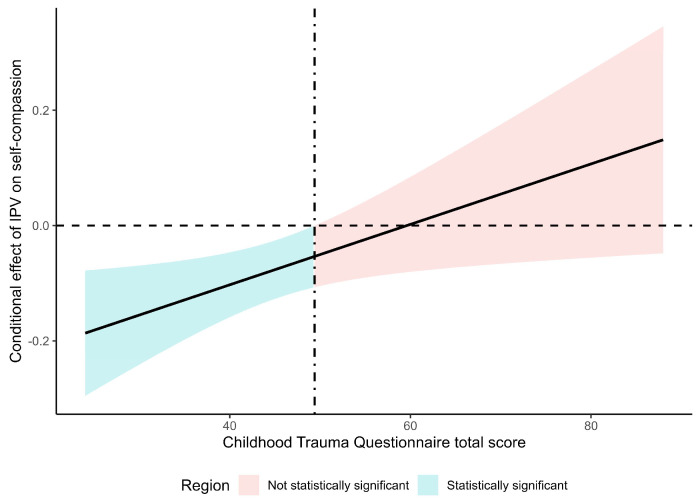
Johnson–Neyman Region of Significance for the Conditional Association Between Intimate Partner Violence and Self-Compassion. The solid line represents the conditional effect of the Composite Abuse Scale total score on self-compassion across the observed range of Childhood Trauma Questionnaire total scores. Shaded areas represent 95% confidence intervals, the horizontal dashed line indicates a null effect, and the vertical dot-dashed line marks the Johnson–Neyman transition point at which the conditional association changes from statistically significant to non-significant.

Given the cross-sectional design, these analyses estimate conditional indirect associations and do not establish temporal mediation. Moderated mediation was examined using a regression-based conditional process model to evaluate whether the associations between IPV and MWB, via SC and PD, varied across levels of CT, adjusting for age, income, education, and employment status (see Table 5 and Table 6, and Figure 1). The interaction between IPV and CT was significant in the model predicting SC, whereas no corresponding interaction effect was observed in the model predicting PD. To further characterize the significant IPV × CT interaction in the SC model, simple slope analyses were conducted at low, mean, and high levels of CT, and the conditional association was additionally examined using the Johnson–Neyman technique (Figure 2 and Figure 3).

Conditional associations between IPV and SC differed across CT levels. At low CT (−1 SD), IPV was negatively associated with SC (B = −0.1570, SE = 0.0443, *p* < 0.001, 95% CI (−0.2440, −0.0698)). This association was weaker at the mean level of CT (B = −0.0901, SE = 0.0267, *p* < 0.001, 95% CI (−0.1430, −0.0377)) and was non-significant at high CT (+1 SD; B = −0.0233, SE = 0.0338, *p* = 0.491, 95% CI (−0.0899, 0.0432)). Standardized conditional coefficients showed the same gradient (β = −0.287, −0.165, and −0.043, respectively; Table 6).

As illustrated in Figure 2, the negative association between IPV and SC progressively weakened as CT increased. The Johnson–Neyman analysis further identified the range of CT scores within which the conditional association between IPV and SC remained statistically significant (Figure 3). Conditional indirect associations between IPV and MWB were observed through SC, PD, and their serial combination, with magnitudes varying across CT levels (Table 6). At low CT, the indirect association via SC was −0.0492 (BootSE = 0.0131, 95% CI (−0.0751, −0.0242)), representing 49.4% of the total association. The indirect association via PD was −0.0211 (BootSE = 0.0076, 95% CI (−0.0386, −0.0092], 21.2%)), and the serial SC–PD pathway was −0.0083 (BootSE = 0.0031, 95% CI (−0.0155, −0.0032), 8.3%)). The total indirect effect at low CT was −0.0785 (BootSE = 0.0159, 95% CI (−0.1108, −0.0478)), accounting for 78.9% of the total association.

At the mean level of CT, indirect associations through SC (B = −0.0282, BootSE = 0.0085, 95% CI (−0.0461, −0.0126)), PD (B = −0.0182, BootSE = 0.0059, 95% CI (−0.0319, −0.0090)), and the serial pathway (B = −0.0047, BootSE = 0.0020, 95% CI (−0.0094, −0.0016)) accounted for 39.1%, 25.3%, and 6.6% of the total association, respectively. The total indirect effect at the mean level of CT was −0.0512 (BootSE = 0.0110, 95% CI (−0.0750, −0.0319)), accounting for 70.9% of the total association.

At high CT, the total indirect effect was smaller but remained significant (B = −0.0239, BootSE = 0.0116, 95% CI (−0.0505, −0.0044)), accounting for 53.3% of the total association. At this level, the indirect association via PD remained significant and represented the largest component of the indirect association (B = −0.0154, BootSE = 0.0058, 95% CI (−0.0292, −0.0065), 34.3%). In contrast, the indirect association via SC and the serial SC–PD pathway were attenuated and non-significant.

## 4. Discussion

The present study examined the associations between IPV, CT, SC, PD and MWB in a clinical sample of women presenting to psychiatric outpatient services. By modeling PD and MWB concurrently and considering SC as a key psychological resource within this framework, the findings contribute to a more nuanced understanding of IPV-related mental health in clinical contexts. The results highlight distinct patterns of association across childhood trauma and key psychological constructs. They offer insight into how relational adversity is reflected differently in self-related resources, PD, and MWB.

Hierarchical regression analyses indicated that, in both models, SC subcomponents accounted for the largest proportion of explained variance in MWB. This pattern is consistent with the first hypothesis. When CT and IPV were entered in the first block, the amount of explained variance remained relatively limited; however, the inclusion of SC variables resulted in a substantial increase in model explanatory power. These findings suggest that SC subscales were strongly associated with MWB and explained substantial additional variance beyond CT, IPV, and sociodemographic variables. Previous hierarchical regression and mediation studies have similarly shown that SC functions as a distinct mechanism supporting emotion regulation, functioning, and psychological well-being beyond the burden of trauma-related symptoms [49,50,51].

Our second hypothesis was supported by the serial mediation findings. These findings suggest that the association between IPV and MWB may be better understood when multiple, interrelated psychological dimensions are considered together. Examining these dimensions in isolation may provide an incomplete picture. While prior research has documented bivariate associations between IPV, SC, PD and MWB [4,10,13,52,53], the current findings indicate that these constructs are interrelated within the same relational context. In this framework, IPV-related experiences appear to be concurrently associated with self-related psychological resources and distress-related symptoms, both of which were also associated with MWB. The presence of serial indirect associations involving SC and PD highlights the value of integrative models that account for both resource-oriented and vulnerability-related aspects of mental health. Nevertheless, these serial indirect associations should be interpreted as cross-sectional associations rather than evidence of directional pathways, as lower MWB, current depressive or anxiety symptoms, and unmeasured factors such as personality, social support, economic dependence, or relationship context may also contribute to the observed pattern. Taken together, these findings are consistent with perspectives emphasizing that MWB cannot be inferred solely from symptom burden. They also highlight the importance of examining self-related constructs alongside distress when interpreting IPV-related mental health correlates in clinical samples.

Previous mediation studies have mainly focused on CT, identifying SC as a self-related resource linking early adversity to both PD and MWB [29,30]. The present serial mediation findings extend this work by suggesting that similar self-related processes may also be relevant in the context of ongoing relational adversity such as IPV. Although IPV differs from CT in timing and relational dynamics, both involve interpersonal threat and compromised emotional safety. Accordingly, the observed serial associations involving SC and PD are consistent with trauma-focused mediation models while extending them to adult relational contexts.

The third hypothesis was supported. Moderation analyses further indicated that CT was associated with the nature of the relationship between IPV and SC, whereas no comparable moderating effect was observed for the relationship between IPV and PD. Specifically, IPV was associated with lower SC at low and mean levels of CT, but not at high levels. Because SC is considered a self-regulatory resource rooted in early relational experiences [21,54] and CT has been consistently associated with lower SC [49,55], one possible interpretation is that women with high CT may already have had relatively low and less variable SC scores, making an additional association with current IPV more difficult to detect. This pattern could also be consistent with a floor effect or with statistical overshadowing by cumulative developmental adversity, whereby the enduring effects of early trauma may reduce the detectable unique association between current relational violence and self-related functioning. Trauma-related avoidance, dissociative coping, or differential reporting may represent other possible explanations. However, these interpretations remain speculative because the proposed mechanisms and the distribution of SC across CT levels were not directly examined. Accordingly, the nonsignificant association at high CT should not be taken to indicate that IPV is psychologically unimportant, but rather that its association with SC may be less readily observable in this subgroup.

The fourth hypothesis was not supported. The absence of a moderating effect of CT on the IPV–PD association may reflect the broad and relatively proximal stress-response nature of psychological distress. Ongoing IPV may be associated with depressive symptoms, anxiety, and general distress through current experiences of threat, fear, uncertainty, and chronic interpersonal stress, such that the strength of this association may be less dependent on developmental trauma history. Consistent with this interpretation, a large body of literature has consistently demonstrated the association between IPV and depressive symptoms, anxiety, and general psychological distress across diverse populations [56,57]. Within this framework, CT may be better conceptualized not as a direct determinant of IPV-related psychological outcomes but as a contextual factor associated with interindividual differences when considered alongside resource-based processes such as SC. Although the cross-sectional design precludes conclusions regarding directionality, the findings highlight the value of distinguishing between symptom-based outcomes and self-related regulatory processes when examining the psychological correlates of IPV.

### Limitations and Future Perspectives

Several limitations should be acknowledged in this study. First, the cross-sectional design does not allow for causal or temporal inferences, and the direction of the observed associations cannot be determined. Second, all data were obtained through self-report measures, which may be subject to recall bias, social desirability effects, and common-method bias due to the use of the same measurement method for all main study variables. In addition, IPV may have been underreported because of fear, shame, stigma, safety concerns, or concerns about confidentiality, despite the precautions taken to ensure privacy during data collection. Third, psychiatric diagnoses were based on DSM-5-oriented routine clinical interviews conducted by psychiatrists rather than structured diagnostic interviews, which may have introduced diagnostic variability and limited the comparability of diagnostic classifications across participants. Fourth, the sample consisted of women presenting to psychiatric outpatient services and was characterized by relatively high educational attainment. This may limit the generalizability of the findings to non-clinical populations or to women with different sociodemographic characteristics. In addition, the study was conducted at a single clinical center, which may further limit the representativeness of the sample. Although participants were recruited using a convenience sampling approach, detailed recruitment flow data, including the number of women approached, the number refusing participation, and the number excluded with specific reasons, were not systematically recorded. Therefore, potential selection bias cannot be fully ruled out. The sample was also restricted to married women, and therefore the findings may not be directly applicable to women in other relationship contexts. Moreover, IPV was assessed without detailed differentiation regarding duration, chronicity, specific patterns of exposure, recency, coercive control, perpetrator characteristics, or help-seeking behaviors, which may be relevant for mental health outcomes. The study also did not include measures of social support, which may buffer or modify the associations among IPV, childhood trauma, self-compassion, psychological distress, and mental well-being. Finally, cultural and societal factors related to gender roles and help-seeking behaviors may have influenced the observed associations. In this context, intersectional variables such as migration background, disability status, rural or urban residence, and culturally specific norms were not systematically assessed.

The findings of this study suggest that mental health assessments of women exposed to IPV should not be limited to the evaluation of psychiatric symptom severity alone. Rather, they should also encompass MWB and internal psychological resources such as SC. In practical terms, these findings point toward several concrete directions for psychiatric outpatient settings. Systematic attention to IPV within routine clinical assessment, embedded within a safety-first, trauma-informed framework, has been highlighted as a key component of mental health service response to IPV [28]. Such an approach would involve not only sensitive inquiry about IPV exposure but also clear pathways for referral to specialized support services, given that psychiatric care alone may not adequately address the safety-related and structural dimensions of IPV [28]. The moderating role of CT in the IPV–SC association further suggests that assessing trauma history alongside SC levels may provide additional clinically relevant information about individual differences among women exposed to IPV. In this regard, preliminary pilot work on group compassion-focused therapy with survivors of IPV and gender-based violence provides initial evidence for the feasibility of such interventions in this population [25], while broader meta-analytic evidence indicates that SC-focused interventions may be associated with improvements in psychological outcomes across various clinical and non-clinical samples [58]. Taken together, these considerations suggest that trauma-informed, safety-oriented assessment combined with the potential integration of SC-focused components may represent a direction worth exploring in future clinical and interventional work with IPV-exposed women. It should be noted, however, that SC-focused approaches are not intended as a substitute for safety planning or structural support; rather, they may be considered a complementary element within a broader, safety-oriented model of care.

Future research should employ longitudinal designs to clarify the direction and temporal sequencing of the associations observed in this study. The finding that CT moderated the IPV–SC association but not the IPV–PD association suggests that these constructs may show different patterns of association, warranting closer theoretical examination. In this context, greater attention to interpersonal and emotion regulation processes related to SC may be particularly informative for future models. Additionally, studies conducted in diverse clinical and sociocultural samples would help clarify the extent to which the present findings generalize beyond the current sample.

## 5. Conclusions

In conclusion, this study underscores the importance of examining IPV within a broader mental health framework that includes both PD and MWB in clinical populations. By considering SC and CT alongside IPV, the findings highlight the value of attending to self-related psychological constructs and developmental context when interpreting IPV-related mental health correlates. Notably, SC subscales accounted for the largest increment in explained variance in MWB across both regression models (ΔR^2^ = 0.256 and 0.294, both *p* < 0.001), beyond the contributions of IPV, CT, and sociodemographic covariates. However, because the cross-sectional design precludes causal inference, longitudinal studies are needed before these associations can be interpreted as reflecting underlying mechanisms. Taken together, these results support a more integrative and nuanced approach to understanding women’s mental health in psychiatric settings.

## Figures and Tables

**Table 1:** Sociodemographic and Clinical Characteristics of the Participants.

Variable	Category	*n*/Mean ± SD	%/Min–Max
Age (years)	—	39.67 ± 9.48	20–65
Marriage duration (years)	—	15.90 ± 10.24	1–47
Age at marriage (years)	—	23.68 ± 5.28	15–52
Education level	Primary	55	16.2
	Secondary	57	16.8
	High school	111	32.6
	Associate degree	46	13.5
	Bachelor’s degree	71	20.9
Lifetime psychiatric diagnosis	No	92	27.1
	Yes	248	72.9
Current psychotropic medication use	No	156	45.9
	Yes	184	54.1
DSM-5-based clinical diagnosis at assessment	Major depressive disorder	133	39.1
	Anxiety disorders	105	30.9
	Subthreshold depressive and/or anxiety symptoms	61	17.9
	Obsessive–compulsive disorder	20	5.9
	Trauma and stressor related disorders	14	4.1
	Somatic symptom disorder	7	2.1
Employment status	No	224	65.9
	Yes	116	34.1
Extended family living	No	292	85.9
	Yes	45	13.2
	Other	3	0.9
Income level	Low	29	8.5
	Low to moderate	120	35.3
	Moderate	109	32.1
	High	82	24.1
Family history of psychiatric disorder	No	244	72.0
	Yes	95	28.0
Current smoking	No	197	57.9
	Yes	143	42.1
Current alcohol consumption	No	287	84.4
	Yes	53	15.6
History of suicide attempt	Yes	57	16.8
	No	283	83.2
Stressful life events (last 3 months)	No	153	45.0
	Yes	187	55.0
CAS (Severe Combined) Category	No	259	76.2
	Yes	81	23.8
CAS (Physical Abuse) Category	No	215	63.2
	Yes	125	36.8
CAS (Emotional Abuse) Category	No	166	48.8
	Yes	174	51.2
CAS (Harassment) Category	No	301	88.5
	Yes	39	11.5
CAS (Total) Category (≥3)	No	145	42.6
	Yes	195	57.4
CAS (Total) Category (≥7)	No	301	88.5
	Yes	39	11.5

**Table 2:** Internal Consistency and Descriptive Statistics of Study Scales and Subscales.

Measurements	Cronbach’s α	Mean ± SD	Min–Max
CTQ—Emotional abuse	0.758	8.41 ± 3.97	5–25
CTQ—Emotional neglect	0.821	13.84 ± 5.40	5–25
CTQ—Physical abuse	0.791	6.03 ± 2.43	5–19
CTQ—Physical neglect	0.704	7.63 ± 2.80	5–17
CTQ—Sexual abuse	0.909	6.50 ± 3.61	5–25
CTQ Total	0.798	42.41 ± 12.75	25–88
CAS—Severe combined abuse	0.709	0.99 ± 2.78	0–26
CAS—Physical abuse	0.940	2.68 ± 5.15	0–35
CAS—Emotional abuse	0.869	6.13 ± 8.03	0–47
CAS—Harassment	0.809	0.62 ± 2.20	0–20
CAS Total	0.936	10.42 ± 15.68	0–122
SCS—Self-kindness	0.871	4.77 ± 2.28	2–10
SCS—Self-judgment	0.696	7.15 ± 2.24	2–10
SCS—Common humanity	0.744	5.31 ± 2.10	2–10
SCS—Isolation	0.706	6.88 ± 2.39	2–10
SCS—Mindfulness	0.765	5.28 ± 2.10	2–10
SCS—Over-identification	0.831	6.40 ± 2.59	2–10
SCS Total	0.812	35.79 ± 8.58	16–56
Mental well-being (Warwick-Edinburgh Mental Well-being Scale Short Form)	0.839	22.59 ± 5.82	9–35
Psychological distress (PHQ-4)	0.789	7.08 ± 2.95	0–12

CTQ = Childhood Trauma Questionnaire; CAS = Composite Abuse Scale; SCS = Self-compassion scale; SD = Standard Deviation.

**Table 3:** Bivariate Correlations Among the Main Study Variables.

	MWB	SCS Total	PHQ-4	CTQ Total
1.SCS Total	0.556 **			
2.PHQ-4	−0.427 **	−0.417 **		
3.CTQ Total	−0.230 **	−0.239 **	0.076	
4.CAS Total	−0.190 **	−0.167 **	0.258 **	0.243 **

Values represent Pearson correlation coefficients. MWB = mental well-being; SCS = self-compassion; PHQ-4 = psychological distress; CTQ = Childhood Trauma Questionnaire; CAS = Composite Abuse Scale. ** *p* < 0.01.

**Table 4:** Hierarchical Regression Analyses Predicting Mental Well-Being.

Model 1	B	SE	β	t	95% CI for B	*p*
Block 1						
CTQ—Emotional abuse	−0.405	0.104	−0.277	−3.906	(−0.610, −0.201)	<0.001
CTQ—Emotional neglect	−0.116	0.068	−0.108	−1.707	(−0.251, 0.018)	0.089
CTQ—Physical abuse	0.215	0.155	0.090	1.386	(−0.090, 0.520)	0.167
CTQ—Physical neglect	0.099	0.136	0.048	0.733	(−0.168, 0.366)	0.464
CTQ—Sexual abuse	−0.040	0.091	−0.025	−0.440	(−0.218, 0.138)	0.660
Block 1 change ΔR^2^ = 0.074, *p* < 0.001						
Block 2						
CTQ—Emotional abuse	−0.135	0.095	−0.092	−1.423	(−0.322, 0.052)	0.156
CTQ—Emotional neglect	−0.077	0.060	−0.071	−1.279	(−0.195, 0.041)	0.202
CTQ—Physical abuse	0.083	0.134	0.035	0.620	(−0.180, 0.346)	0.536
CTQ—Physical neglect	0.105	0.116	0.050	0.906	(−0.123, 0.333)	0.366
CTQ—Sexual abuse	−0.056	0.078	−0.035	−0.725	(−0.209, 0.096)	0.469
SCS—Self-kindness	0.627	0.141	0.246	4.458	(0.350, 0.903)	<0.001
SCS—Self-judgment	−0.070	0.137	−0.027	−0.509	(−0.340, 0.200)	0.611
SCS—Common humanity	0.119	0.141	0.043	0.840	(−0.159, 0.397)	0.401
SCS—Isolation	−0.639	0.136	−0.262	−4.705	(−0.906,−0.372)	<0.001
SCS—Mindfulness	0.526	0.153	0.190	3.428	(0.224, 0.827)	0.001
SCS—Over-identification	−0.023	0.111	−0.010	−0.209	(−0.242,0.195)	0.835
Block 2 change ΔR^2^ = 0.256, *p* < 0.001						
Model 2						
Block 1						
CAS—Severe combined abuse	−0.254	0.183	−0.121	−1.383	(−0.615, 0.107)	0.168
CAS—Physical abuse	0.028	0.083	0.025	0.337	(−0.136, 0.192)	0.736
CAS—Emotional abuse	−0.078	0.057	−0.108	−1.362	(−0.191, 0.035)	0.174
CAS—Harassment	0.014	0.216	0.005	0.067	(−0.411, 0.440)	0.947
Block 1 change ΔR^2^ = 0.033, *p* = 0.019						
Block 2						
CAS—Severe combined abuse	−0.129	0.155	−0.062	−0.834	(−0.434, 0.176)	0.405
CAS—Physical abuse	−0.010	0.071	−0.009	−0.141	(−0.149, 0.129)	0.888
CAS—Emotional abuse	−0.019	0.049	−0.026	−0.379	(−0.116, 0.078)	0.705
CAS—Harassment	−0.034	0.182	−0.013	−0.187	(−0.393, 0.325)	0.851
SCS—Self-kindness	0.671	0.139	0.263	4.809	(0.396, 0.945)	<0.001
SCS—Self-judgment	−0.072	0.138	−0.028	−0.525	(−0.344,0.199)	0.600
SCS—Common humanity	0.151	0.141	0.055	1.072	(−0.126, 0.429)	0.284
SCS—Isolation	−0.632	0.134	−0.260	−4.706	(−0.897,−0.368)	<0.001
SCS—Mindfulness	0.515	0.153	0.186	3.369	(0.214, 0.815)	0.001
SCS—Over-identification	−0.036	0.112	−0.016	−0.323	(−0.257,0.184)	0.747
Block 2 change ΔR^2^ = 0.294, *p* < 0.001						

The dependent variable was mental well-being. B = unstandardized regression coefficient; SE = standard error; β = standardized regression coefficient; CI = confidence interval; CTQ = Childhood Trauma Questionnaire; CAS = Composite Abuse Scale; SCS = Self-Compassion Scale. In both models, self-compassion subscales were entered in Block 2. ΔR^2^ indicates the change in explained variance associated with each block. All models adjusted for age, income, education, and employment status.

**Table 5:** Regression Equations for the Moderated Mediation Path Model.

Outcome	Predictor	β	B	SE	t	*p*	95% CI
SCS	CAS	−0.165	−0.0901	0.0267	−3.38	<0.001	(−0.1430, −0.0377)
	CTQ	−0.230	−0.1548	0.0360	−4.30	<0.001	(−0.2260, −0.0839)
	CAS × CTQ	0.178	0.0052	0.0023	2.30	0.022	(0.0008, 0.0097)
Model fit: R^2^ = 0.175; F(7, 332) = 10.00; *p* < 0.001							
PHQ	SCS	−0.345	−0.1185	0.0190	−6.24	<0.001	(−0.1560, −0.0811)
	CAS	0.219	0.0411	0.0097	4.24	<0.001	(0.0220, 0.0602)
	CTQ	−0.057	−0.0131	0.0129	−1.01	0.313	(−0.0385, 0.0124)
	CAS × CTQ	−0.050	−0.0005	0.0006	−0.82	0.411	(−0.0017, 0.0007)
Model fit: R^2^ = 0.239; F(8, 331) = 13.00; *p* < 0.001							
MWB	SCS	0.462	0.3132	0.0337	9.30	<0.001	(0.2470, 0.3790)
	PHQ	−0.225	−0.4437	0.0945	−4.69	<0.001	(−0.6300, −0.2580)
	CAS	−0.057	−0.0210	0.0188	−1.12	0.265	(−0.0580, 0.0160)
Model fit: R^2^ = 0.370; F(7, 332) = 27.90; *p* < 0.001							

β = standardized regression coefficient; B = unstandardized coefficient; SE = standard error; CI = confidence interval. CAS = Composite Abuse Scale total score; CTQ = Childhood Trauma Questionnaire total score; SCS = self-compassion total score; PHQ = psychological distress (PHQ-4); MWB = mental well-being. Interaction terms represent the product of mean-centered predictors. Model fit indices (R^2^, F statistics, and *p* values) are reported for each outcome. All models adjusted for age, income, education, and employment status.

**Table 6:** Conditional Direct and Indirect Effects in the Moderated Serial Mediation Model.

Path	CTQ Level	β	Effect (B)	SE/BootSE	t	*p*	95% CI/Boot CI
Direct: CAS → SCS	−1 SD	−0.287	−0.1570	0.0443	−3.54	<0.001	(−0.2440, −0.0698)
	Mean	−0.165	−0.0901	0.0267	−3.38	<0.001	(−0.1430, −0.0377)
	+1 SD	−0.043	−0.0233	0.0338	−0.69	0.491	(−0.0899, 0.0432)
Indirect: CAS → SCS → MWB	−1 SD	—	−0.0492	0.0131	—	—	(−0.0751, −0.0242)
	Mean	—	−0.0282	0.0085	—	—	(−0.0461, −0.0126)
	+1 SD	—	−0.0073	0.0092	—	—	(−0.0271, 0.0095)
Indirect: CAS → PHQ → MWB	−1 SD	—	−0.0211	0.0076	—	—	(−0.0386, −0.0092)
	Mean	—	−0.0182	0.0059	—	—	(−0.0319, −0.0090)
	+1 SD	—	−0.0154	0.0058	—	—	(−0.0292, −0.0065)
Indirect: CAS → SCS → PHQ → MWB	−1 SD	—	−0.0083	0.0031	—	—	(−0.0155, −0.0032)
	Mean	—	−0.0047	0.0020	—	—	(−0.0094, −0.0016)
	+1 SD	—	−0.0012	0.0017	—	—	(−0.0053, 0.0015)
Total indirect effect	−1 SD	—	−0.0785	0.0159	—	—	(−0.1108, −0.0478)
	Mean	—	−0.0512	0.0110	—	—	(−0.0750, −0.0319)
	+1 SD	—	−0.0239	0.0116	—	—	(−0.0505, −0.0044)

Conditional effects are reported at −1 SD, mean, and +1 SD levels of CTQ. Unstandardized indirect effects are based on bias-corrected bootstrap estimates with 5000 resamples. CAS = Composite Abuse Scale; CTQ = Childhood Trauma Questionnaire; SCS = self-compassion; PHQ = psychological distress (PHQ-4); MWB = Mental well-being.

## Data Availability

The data supporting the findings of this study are not publicly available due to privacy and ethical restrictions, as they contain sensitive information that could compromise the confidentiality of the participants. Data may be made available from the corresponding author, Zeynep Nur Demirok [zeynepnurdemirok@gmail.com], upon reasonable request and subject to applicable ethical and institutional approvals.

## Supplementary Materials

The following supporting information can be downloaded at: https://www.mdpi.com/article/10.3390/jcm15155809/s1. Table S1: Sensitivity Analysis Using Hierarchical Robust Linear Regression; Table S2: Block-Wise Change Statistics Based on Robust Regression; Figure S1: Correlational heatmap displaying the intercorrelations among demographic variables, childhood trauma indicators (CTQ), intimate partner violence measures (CAS), self-compassion dimensions (SC), psychological distress (PHQ), and mental well-being (WB). Cells present Pearson correlation coefficients ® together with corresponding p values. Color intensity reflects the strength and direction of the correlations (red = positive, blue = negative). Only the lower triangular portion of the correlation matrix is shown for clarity.

## Abbreviations

The following abbreviations are used in this manuscript:

MWB	Mental well-being
IPV	Intimate partner violence
CT	Childhood trauma
ACE	Adverse childhood experiences
PD	Psychological distress
SC	Self-compassion
