# Supplementary material for: Intimate Partner Violence and Mental Well-Being Among Married Women Attending Psychiatric Outpatient Clinics: The Roles of Childhood Trauma and Self-Compassion
Source: J Clin Med. 2026 Jul 24;15(15):5809. doi: 10.3390/jcm15155809 (PMC13467478; doi:10.3390/jcm15155809)
Supplement: Supplementary file 1 [file jcm-15-05809-s001.zip › jcm-4416748-supplementary.pdf]

Table S1. Sensitivity Analysis Using Hierarchical Robust Linear Regression

Predictor	b	SE	t	p
Block 1: Covariates				
Age	0.038	0.049	0.77	0.441
Income (high)	−0.019	0.047	−0.39	0.697
High school education or above	0.053	0.048	1.09	0.276
Employment	0.089	0.047	1.87	0.061
Model statistics				
Pseudo-R^2	0.046			
Block 2: Covariates and CAS subscales				
Age	0.036	0.048	0.75	0.453
Income (high)	−0.021	0.047	−0.45	0.653
High school education or above	0.051	0.048	1.06	0.289
Employment	0.091	0.047	1.93	0.054
CAS—Severe combined	−0.047	0.075	−0.62	0.535
CAS—Physical abuse	−0.020	0.064	−0.31	0.756
CAS—Emotional abuse	−0.027	0.069	−0.39	0.697
CAS—Harassment	−0.006	0.070	−0.09	0.928
Model statistics				
Pseudo-R^2	0.080			
ΔPseudo-R^2	0.034			
Block 3: Covariates, CAS subscales, and self-compassion subscales				
Age	0.038	0.049	0.77	0.441
Income (high)	−0.019	0.047	−0.39	0.697
High school education or above	0.053	0.048	1.09	0.276
Employment	0.089	0.047	1.87	0.061
CAS—Severe combined	−0.047	0.075	−0.62	0.535
CAS—Physical abuse	−0.020	0.064	−0.31	0.756
CAS—Emotional abuse	−0.027	0.069	−0.39	0.697
CAS—Harassment	−0.006	0.070	−0.09	0.928
SCS—Self-kindness	0.260	0.056	4.65	< .001
SCS—Self-judgment	0.049	0.054	0.91	0.363
SCS—Common humanity	0.070	0.052	1.34	0.180
SCS—Isolation	0.244	0.057	4.30	< .001
SCS—Mindfulness	0.223	0.057	3.91	< .001
SCS—Over-identification	−0.017	0.051	−0.32	0.749
Model statistics				
Pseudo-R^2	0.373			
ΔPseudo-R^2	0.293			

CAS = Childhood Abuse Scale; SCS = Self-Compassion. The sensitivity analysis was conducted using the LMROB robust regression estimator. ΔPseudo-R^2 represents the increase in pseudo-R^2 relative to the preceding block.

Table S2. Block-Wise Change Statistics Based on Robust Regression

Model comparison	Initial R^2	Final R^2	ΔR^2	F change	p
Block 1 → Block 2	.046	.080	.034	3.41	< 0.01
Block 2 → Block 3	.080	.373	.293	24.80	< 0.001

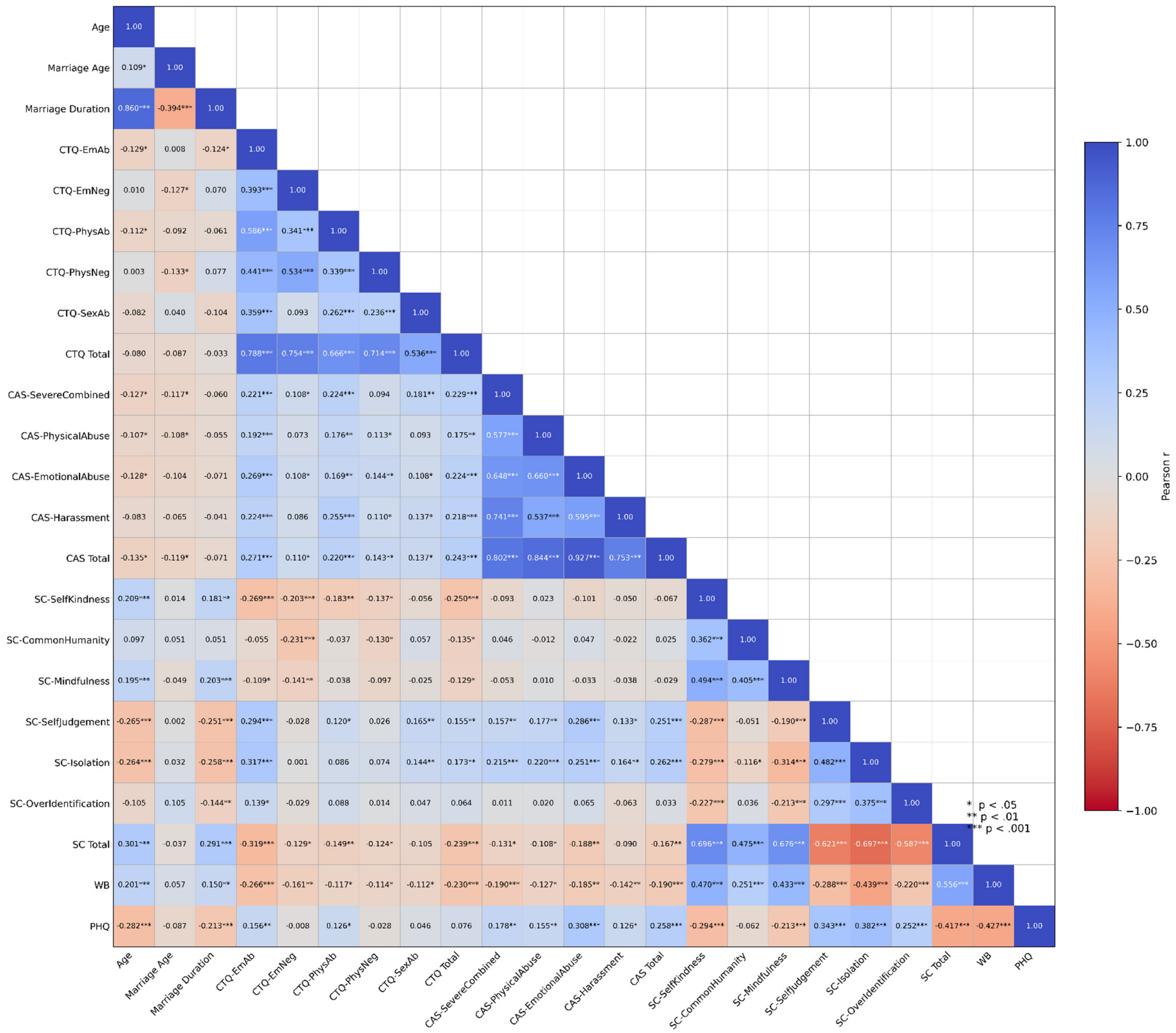

Age
Marriage Age
Marriage Duration
CTQ-EmAb
CTQ-EmNeg
CTQ-PhysAb
CTQ-PhysNeg
CTQ-SexAb
CTQ Total
CAS-SevereCombined
CAS-PhysicalAbuse
CAS-EmotionalAbuse
CAS-Harassment
CAS Total
SC-SelfKindness
SC-CommonHumanity
SC-Mindfulness
SC-SelfJudgement
SC-Isolation
SC-OverIdentification
SC Total
WB
PHQ
Pearson r
1.00
0.75
0.50
0.25
0.00
−0.25
−0.50
−0.75
−1.00
* p < .05
** p < .01
*** p < .001

Figure S1. Correlational heatmap displaying the intercorrelations among demographic variables, childhood trauma indicators (CTQ), intimate partner violence measures (CAS), self-compassion dimensions (SC), psychological distress (PHQ), and mental well-being (WB). Cells present Pearson correlation coefficients ® together with corresponding *p* values. Color intensity reflects the strength and direction of the correlations (red = positive, blue = negative). Only the lower triangular portion of the correlation matrix is shown for clarity.
